# Baroreflex Impairment Precedes Cardiometabolic Dysfunction in an Experimental Model of Metabolic Syndrome: Role of Inflammation and Oxidative Stress

**DOI:** 10.1038/s41598-018-26816-4

**Published:** 2018-06-05

**Authors:** Nathalia Bernardes, Danielle da Silva Dias, Filipe Fernandes Stoyell-Conti, Janaina de Oliveira Brito-Monzani, Christiane Malfitano, Elia Garcia Caldini, Luis Ulloa, Susana Francisca Llesuy, Maria-Cláudia Irigoyen, Kátia De Angelis

**Affiliations:** 1 Laboratory of Translational Physiology, Universidade Nove de Julho (UNINOVE), São Paulo, SP Brazil; 2 Laboratory of Physiology and Metabolism, Cidade de Sao Paulo University (UNICID), São Paulo, SP Brazil; 3 Federal University of Maranhao, São Luís, MA Brazil; 4 Department of Health Sciences, Federal University of Lavras (UFLA), Lavras, MG, Brazil; 5 Hypertension Unit, Heart Institute (InCor), University of Sao Paulo (USP), São Paulo, SP Brazil; 6 Department of Surgery, Center of Immunology and Inflammation, Rutgers-New Jersey Medical School, Rutgers University, Newark, NJ USA; 7 Department of General and Inorganic Chemistry, University of Buenos Aires, Buenos Aires, BA Argentina; 8 Departament of Physiology, Federal University of São Paulo (UNIFESP), São Paulo, SP Brazil

## Abstract

This study analyzes whether autonomic dysfunction precedes cardiometabolic alterations in spontaneously hypertensive rats (SHR) with fructose overload. Animals were randomly distributed into three groups: control, hypertensive and hypertensive with fructose overload. Fructose overload (100 g/L) was initiated at 30 days old, and the animals (n = 6/group/time) were evaluated after 7, 15, 30 and 60 days of fructose consumption. Fructose consumption reduced baroreflex sensitivity by day 7, and still induced a progressive reduction in baroreflex sensitivity over the time. Fructose consumption also increased TNFα and IL-6 levels in the adipose tissue and IL-1β levels in the spleen at days 15 and 30. Fructose consumption also reduced plasmatic nitrites (day 15 and 30) and superoxide dismutase activity (day 15 and 60), but increased hydrogen peroxide (day 30 and 60), lipid peroxidation and protein oxidation (day 60). Fructose consumption increased arterial pressure at day 30 (8%) and 60 (11%). Fructose consumption also induced a late insulin resistance at day 60, but did not affect glucose levels. In conclusion, the results show that baroreflex sensitivity impairment precedes inflammatory and oxidative stress disorders, probably by inducing hemodynamic and metabolic dysfunctions observed in metabolic syndrome.

## Introduction

Modern medicine is challenged by an increased prevalence of cardiometabolic disorders including diabetes, hypertension, dyslipidemia and metabolic syndrome^1–4^. These disorders represent a major social and clinical burden inducing morbidity and mortality and reducing quality and life expectancy^5^. An increasing number of investigators are studying the underling mechanisms in order to design preventive and therapeutic strategies for these epidemic disorders.

Recent studies correlated the cardiovascular and autonomic dysfunction with the metabolic syndrome in both experimental and clinical settings^6–9^. Van Gaal *et al*.^9^ postulated that visceral obesity is a common pathway in these disorders, and increases the risk of diabetes and cardiovascular disorders by conventional mechanisms (dyslipidemia, hypertension and glucose dysmetabolism) and by the release of inflammatory mediators^9–11^. We reasoned that a third possibility is that this pathological pattern may also represent an autonomic dysfunction, as the autonomic nervous system modulates inflammatory responses, oxidative stress and the cardiovascular system^10,11^. We tested our hypothesis in spontaneously hypertensive rats (SHR) with fructose overload to induce cardiometabolic dysfunctions^7,12–14^. Fructose consumption has dramatically increased in Western society due to the use of cheap processed products, the expansion of fast-food chains and unfavorable lifestyle changes^3,15^. This dramatic increase in fructose consumption is thought to contribute to the current prevalence of cardiometabolic disorders, such as overweight, dyslipidemia, insulin resistance and hypertension^3,5,15,16^. We previously reported that eight weeks of fructose overload induced cardiovascular alterations associated with autonomic dysfunctions as well as inflammation and oxidative stress^7,8,12–14^. We reported that high sympathetic modulation of blood vessels and heart rate preceded metabolic dysfunction in normotensive mice with fructose overload in drinking water^12^. However, it is still unknown the time course of these events and whether autonomic alterations, evaluated by baroreflex sensitivity, precedes cardiovascular and metabolic dysfunction or they are a consequence. We hypothesized that autonomic dysfunction triggers an increase in both inflammation and oxidative stress, promoting cardiometabolic disorders. This study analyzes whether autonomic alterations, evaluated by baroreflex sensitivity, precedes cardiovascular and metabolic dysfunction as well as the inflammatory and oxidative stress. These studies are critical to establish the common pathway to these disorders in order to design preventive and therapeutic strategies for these epidemic disorders.

## Results

### Fructose overload induced late metabolic, cardiovascular and functional dysfunctions

We first analyzed the effects of the fructose consumption in drinking water (D-fructose, 100 g/L) in SHR rats. Despite all groups presented increase in body weight during the protocol, the group treated with fructose showed higher body weight compared to normotensive (day 7) and hypertensive (days 7 and 15) groups. In addition, fructose consumption induced a statistically significant and lasting increase in blood triglyceride levels from day 15 to 60 as compared to those of normotensive rats. Fructose consumption also induced a late increase in blood triglyceride levels at days 30 and 60, but not at day 15, as compared to those of control hypertensive rats without fructose (Table 1). This increase was specific as compared to other metabolites as fructose consumption did not affect blood glucose levels even when the animals were analyzed after 60 days of treatment. Despite the lack of changes on blood glucose levels, fructose overload increased plasma insulin levels. We noticed that the control hypertensive rats without fructose have slightly higher plasma insulin levels that became significantly higher than those of control normotensive rats at day 60. Fructose consumption in hypertensive animals did not induce a significant effect as compared to the control hypertensive animals without fructose, but significantly increased plasma insulin levels at day 30 and 60 when compared to control normotensive rats. However, these changes in insulin did not correlate with changes in the glucose kinetics and the serum glucose removal rate (KITT). All animals have similar serum glucose removal rate, but fructose consumption significantly decreased the KITT values at day 60 as compared to both normotensive and hypertensive animals **(**Table 1**)**.

**Table 1:** Metabolic, hemodynamic and functional capacity evaluations in control (C), hypertensive (H) and hypertensive + fructose overload (HF) groups at 7, 15, 30 and 60 days of protocol.

	7 days	15 days	30 days	60 days
Body weight (g)				
C	88 ± 4.4	154 ± 3.3^§^	203 ± 4.0^†^	288 ± 12.3^‡^
H	71 ± 4.4^*^	94 ± 4.3^§,*^	176 ± 1.6^†,*^	259 ± 5.6^‡,*^
HF	95 ± 2.9^*,#^	115 ± 3.6^§,*,#^	172 ± 8.2^†,*^	258 ± 4.8^‡,*^
Triglycerides (mg/dL)				
C	107 ± 5.0	109 ± 5.5	109 ± 4.1	107 ± 5.9
H	107 ± 3.6	115 ± 1.9	107 ± 5.7	105 ± 5.3
HF	124 ± 6.5	136 ± 7.7^*^	135 ± 4.9^*,#^	139 ± 6.4^*,#^
Glucose (mg/dL)				
C	96 ± 2.4	102 ± 1.0	95 ± 4.6	99 ± 2.4
H	103 ± 2.8	96 ± 5.7	95 ± 2.1	98 ± 2.1
HF	105 ± 4.6	109 ± 2.0	107 ± 3.1	105 ± 2.6
Insulin (ng/mL)				
C	0.45 ± 0.1	0.65 ± 0.1	0.68 ± 0.1	0.47 ± 0.06
H	0.84 ± 0.2	1.04 ± 0.3	0.98 ± 0.2	1.44 ± 0.07^*^
HF	1.02 ± 0.1	1.42 ± 0.2	1.76 ± 0.2^*^	1.72 ± 0.2^*^
KITT (mg/dl/%)				
C	4.5 ± 0.2	4.4 ± 0.2	4.3 ± 0.3	3.9 ± 0.2
H	4.7 ± 0.2	4.6 ± 0.3	4.2 ± 0.2	3.9 ± 0.1
HF	5.3 ± 0.3	5.1 ± 0.2	4.3 ± 0.2	3.2 ± 0.2^‡,*,#^
DAP (mmHg)				
C	76 ± 2.1	91 ± 2.2	92 ± 3.0	96 ± 1.4
H	103 ± 2.9^*^	109 ± 1.7^*^	118 ± 3.0^*^	141 ± 4.1^‡,*^
HF	101 ± 3.0^*^	107 ± 3.3^*^	132 ± 3.9^†,*,#^	158 ± 4.2^‡,*,#^
SAP (mmHg)				
C	111 ± 2.7	119 ± 1.8	121 ± 3.6	126 ± 2.1
H	144 ± 3.1^*^	161 ± 2.6^*^	165 ± 4.5^*^	192 ± 4.3^‡,*^
HF	144 ± 3.5^*^	148 ± 1.6^*^	175 ± 3.7^†,*,#^	211 ± 5.9^‡,*,#^
HR (bpm)				
C	405 ± 12.3	358 ± 8.2^§^	338 ± 7.6^§^	335 ± 7.3^§^
H	421 ± 13.9	375 ± 5.1^§^	351 ± 7.1^§^	345 ± 7.5^§^
HF	403 ± 13.2	381 ± 6.2	369 ± 5.1	367 ± 10
Maximal exercise test (min)				
C	14 ± 0.5	12 ± 0.6	14 ± 1.1	13 ± 1.0
H	18 ± 0.4^*^	18 ± 0.8^*^	17 ± 0.6^*^	17 ± 1.1
HF	18 ± 0.9^*^	19 ± 0.8^*^	18 ± 1.0^*^	10 ± 0.9^‡,*,#^

Data are expressed as mean ± SEM. Constant rate for blood glucose disappearance (KITT), diastolic arterial pressure (DAP), systolic arterial pressure (SAP), mean arterial pressure (MAP), heart rate (HR). n = 6 animals/group/time. ^§^p < 0.05 vs. 7 days in the same group; ^†^p < 0.05 vs. 7 and 15 days in the same group; ^‡^p < 0.05 vs. 7, 15 and 30 days in the same group; ^*^p < 0.05 vs. C group in the same time; ^#^p < 0.05 vs. H group in the same time.

**Figure 1:**
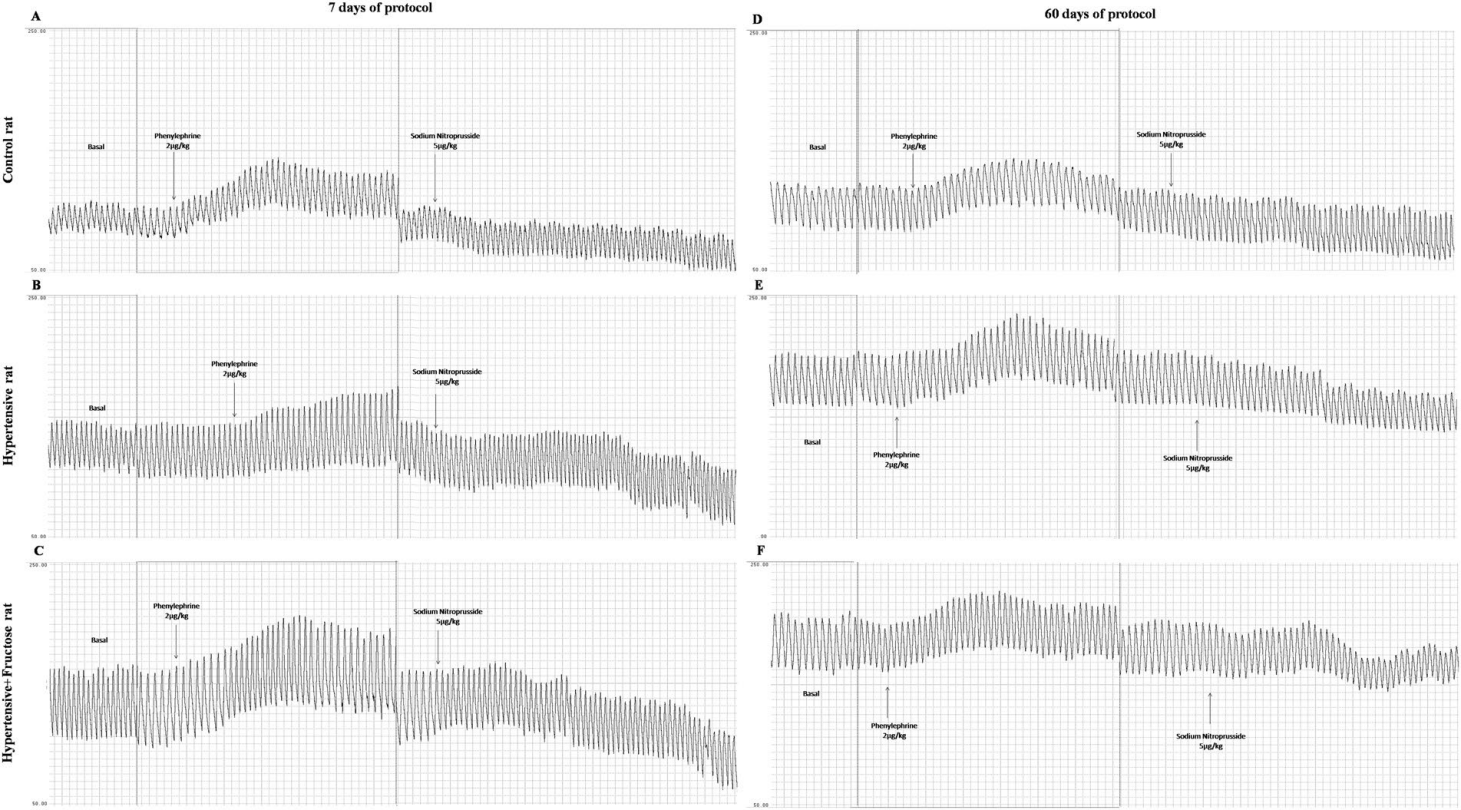
Arterial pressure recording in basal state and after phenylephrine and sodium nitroprusside injections at 7 days of protocol. (**A**) Control rat; (**B**) Hypertensive rat; (**C**) Hypertensive + fructose rat) and at 60 days of protocol. (**D**) Control rat; (**E**) Hypertensive rat and. (**F**) Hypertensive + fructose rat).

Figure 1 shows original recordings of arterial pressure (AP) at basal state and after phenylephrine and sodium nitroprusside to test baroreflex sensitivity in an animal of each evaluated group at 7 and 60 days of protocol.

**Figure 2:**
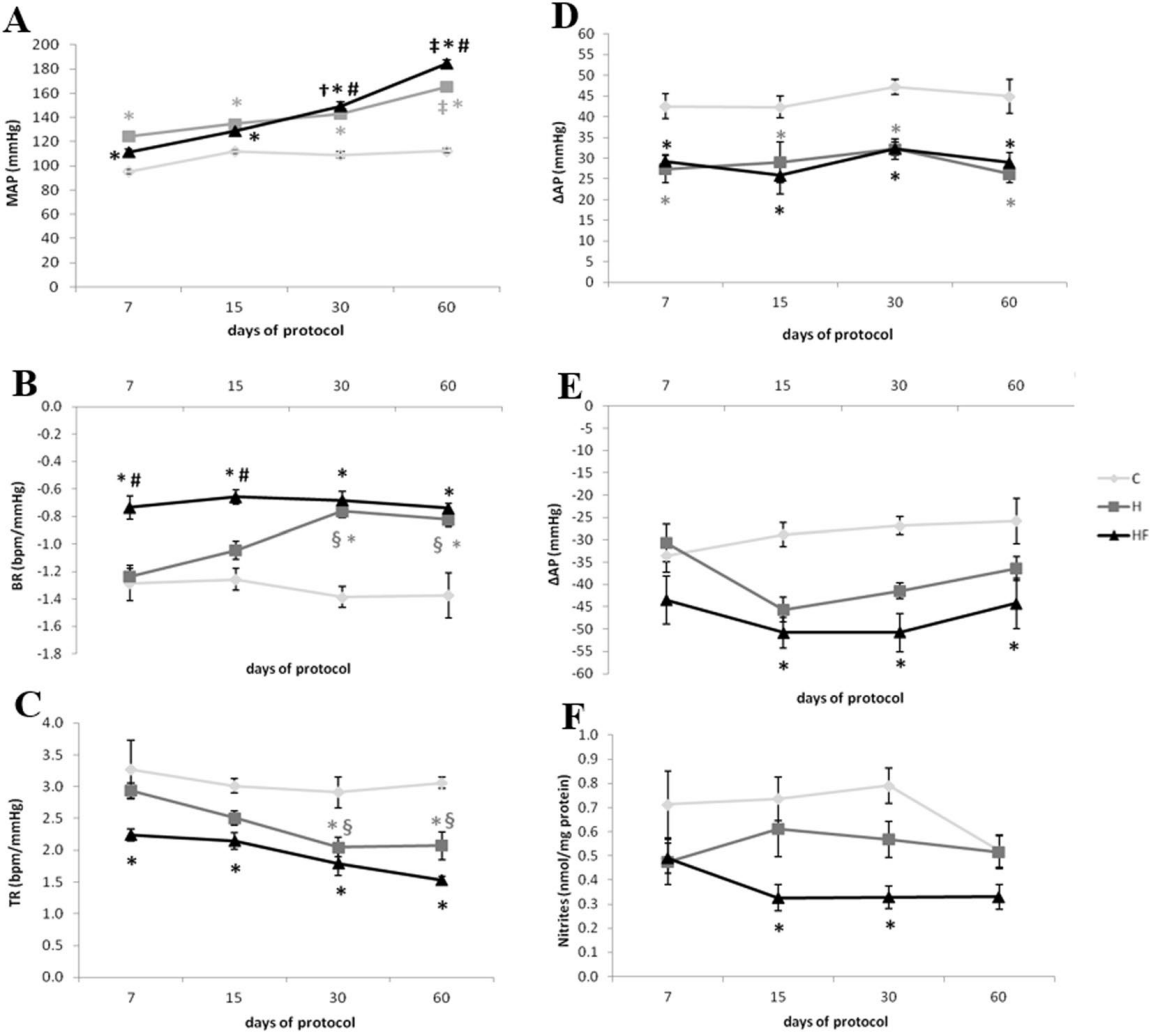
Hemodynamic, autonomic, vascular reactivity and NO bioavailability evaluations. (**A)** Mean arterial pressure (MAP); (**B**) Bradycardic responses (BR) of baroreflex sensitivity; (**C**) Tachycardiac responses (TR) of baroreflex sensitivity; (**D**) Arterial pressure response to 2 μg/kg of phenylephrine; (**E**) Arterial pressure response to 5 μg/kg of sodium nitroprusside and. (**F**) Nitrites in plasma in control (C), hypertensive (H) and hypertensive + fructose overload (HF) groups at 7, 15, 30 and 60 days of protocol. ^§^p < 0.05 vs. 7 days in the same group; ^†^p < 0.05 vs. 7 and 15 days in the same group; ^‡^p < 0.05 vs. 7, 15 and 30 days in the same group; *p < 0.05 vs. C group in the same time; ^#^p < 0.05 vs. H group in the same time. BR: bradycardic responses. TR: tachycardic responses. ∆AP- arterial pressure delta.

Normotensive animals have similar diastolic AP (DAP), systolic AP (SAP) or mean AP (MAP) without significant changes through the protocol from day 7 to 60. Meanwhile, the hypertensive animals showed a gradual increase in DAP, SAP and MAP during the protocol and higher AP values from day 7 through 60 than those of normotensive rats. Fructose consumption induced additional AP increase from day 30 through 60 than those of hypertensive rats (Table 1 and Fig. 2A). By contrast, both normotensive and hypertensive animals have similar heart rate (HR) and kinetic profile with a gradual decrease through the protocol after day 7.

Hypertensive animals with or without fructose treatment significantly run faster in the maximal exercise test than normotensive animals from day 7 to 30. Fructose consumption decreased the physical capacity at day 60 as compared to both normotensive and hypertensive animals without fructose **(**Table 1**)**.

### Baroreflex sensitivity was impaired at day 7 in fructose hypertensive rats

Baroreflex sensitivity decreased in hypertensive animals for bradycardic and tachycardic responses at days 30 and 60 as compared to normotensive animals and to those at day 7. Fructose consumption decreased baroreflex sensitivity at days 7 and 15 as compared to the normotensive (bradycardic and tachycardic responses) and hypertensive animals (bradycardic responses) (Fig. 2B,C).

Regarding vascular response to vasoactive drugs, hypertensive animals with or without fructose have reduced AP response to 2 μg/kg of phenylephrine as compared to normotensive animals (Fig. 2D). Fructose consumption increased the depressor response from day 15 through 60 as compared to normotensive animals (Fig. 2E).

Despite no significant changes in plasma nitrite levels during the protocol in studied groups, fructose consumption in hypertensive animals reduced plasma nitrite (NO bioavailability) levels at day 15 and 30 as compared to those levels of normotensive animals (Fig. 2F).

### Fructose consumption induces inflammatory cytokines in adipose and splenic tissue

**Figure 3:**
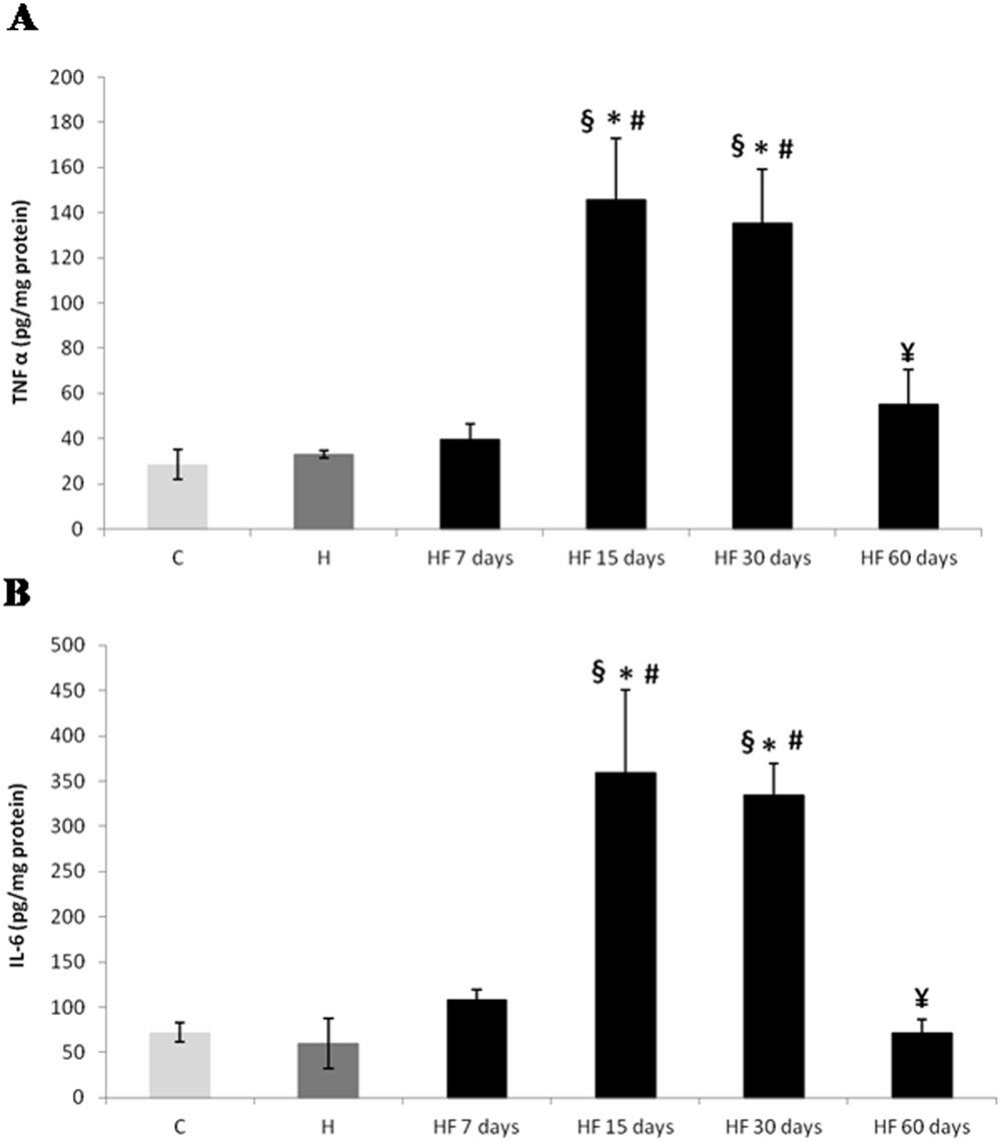
Inflammatory markers in withe adipose tissue. (**A**) TNF-α and. (**B**) IL-6 in control (C), hypertensive (H) and hypertensive + fructose overload (HF) groups at 7 (HF7 days), 15 (HF15 days), 30 (HF30 days) and 60 days of protocol (HF60 days). ^§^p < 0.05 vs. 7 days in the same group; ^¥^p < 0.05 vs. 15 e 30 days in the same group; *p < 0.05 vs. C group in the same time; ^#^p < 0.05 vs. H group in the same time.

One of the most significant effects of fructose consumption was to induce the production of inflammatory cytokines. Fructose consumption temporarily increased the production of IL-1β in the spleen peaking at days 15 and 30 as compared to both normotensive and hypertensive rats without fructose (Table 2). Given the small number of samples with detectable measurements in the cytokine levels in adipose tissue during the protocol of the control normotensive and hypertensive animals without fructose, we represented the result as a single mean (Fig. 3). However, IL-1β in the adipose tissue was higher after fructose consumption at day 15 (63 ± 13 pg/mg protein) than at day 30 (32 ± 7 pg/mg protein) or 60 (5 ± 1 pg/mg protein) as compared to those in normotensive animals (8 ± 2 pg/mg protein). Of note, IL-1β was not detected in the adipose tissue of the hypertensive animals.

**Table 2:** Inflammatory mediators in the spleen of control (C), hypertensive (H) and hypertensive + fructose overload (HF) animals at 7, 15, 30 and 60 days of protocol.

	7 days	15 days	30 days	60 days
TNFα (pg/mg protein)				
C	26 ± 4.7	25 ± 3.4	16 ± 2.3	19 ± 3.4
H	11 ± 1.5	12 ± 0.9	15 ± 1.5	16 ± 1.7
HF	13 ± 1.2	20 ± 5.0	23 ± 2.5	27 ± 1.7
IL-6 (pg/mg protein)				
C	65 ± 5.6	55 ± 5.7	45 ± 5.3	49 ± 3.7
H	58 ± 22.1	39 ± 2.9	50 ± 2.9	49 ± 5.0
HF	42 ± 2.2	55 ± 5.6	48 ± 0.2	34 ± 1.0
IL-1β (pg/mg protein)				
C	64 ± 2.8	82 ± 3.6	49 ± 5.2	48 ± 2.4
H	74 ± 18.0	42 ± 6.7	63 ± 19.0	49 ± 7.9
HF	102 ± 12.1	136 ± 10.1^§,*,#^	110 ± 6.4^*,#^	87 ± 3.5^¶^

Data are expressed as mean ± SEM. TNFα- tumor necrosis factor; IL-6 interleukin-6; IL-1β- interleukin 1 beta. n = 6 animals/group/time. ^§^p < 0.05 vs. 7 days in the same group; ^¶^p < 0.05 vs. 15 days in the same group; ^*^p < 0.05 vs. C group in the same time; ^#^p < 0.05 vs. H group in the same time.

Fructose consumption temporarily increased the production of TNFα and IL-6 in the adipose tissue peaking at days 15 and 30 as compared to both normotensive and hypertensive rats without fructose (Fig. 3A,B). However, there were no significant changes during the protocol in the studied groups, as well as between groups for TNFα and IL-6 in the spleen at all times of measurement (Table 2).

### Fructose consumption induces changes in oxidative stress profile in plasma

**Figure 4:**
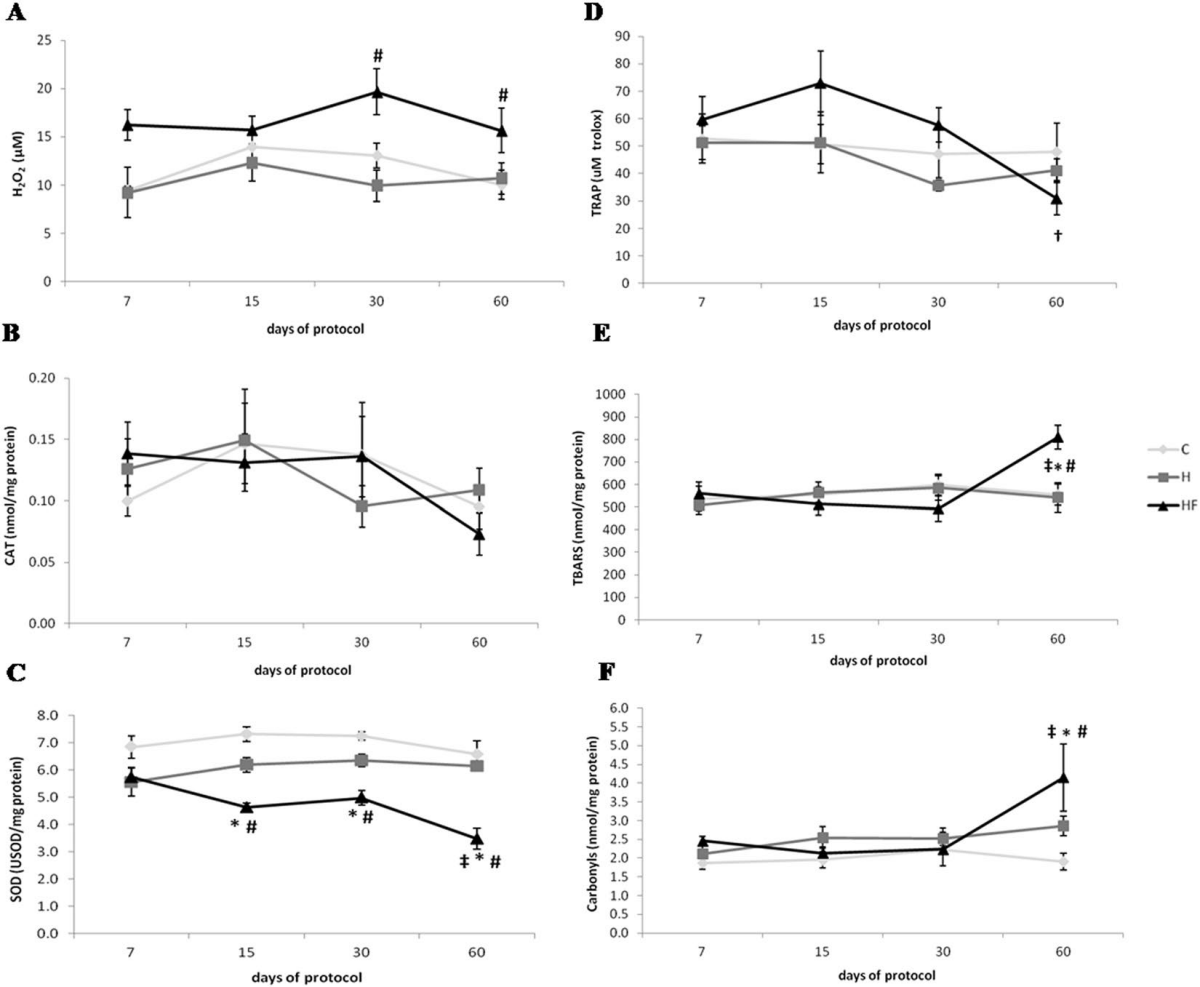
Oxidative stress profile in plasma. (**A**) Hydrogen peroxide; (**B)** Catalase activity (CAT); (**C)** Superoxide dismutase activity (SOD); (**D)** Total antioxidant potential (TRAP); (**E)** Lipid peroxidation (TBARS) and. (**F)** Protein oxidation (Carbonyls) in control (**C**), hypertensive (H) and hypertensive + fructose overload (HF) groups at 7, 15, 30 and 60 days of protocol. ^†^p < 0.05 vs. 7 and 15 days in the same group; ^‡^p < 0.05 vs. 7, 15 and 30 days in the same group; *p < 0.05 vs. C group in the same time; ^#^p < 0.05 vs. H group in the same time.

Regarding reactive oxygen species production, the fructose consumption increased hydrogen peroxide at days 30 and 60 (Fig. 4A). Regarding the antioxidant profile, although no changes were observed in catalase (CAT) activity between the three groups (Fig. 4B), fructose consumption reduced superoxide dismutase (SOD) activity from day 15 to 60. SOD activity was also reduced with fructose consumption at days 15 and 60 when compared to those at day 7 (Fig. 4C). Fructose consumption also impaired non-enzymatic antioxidant capacity, evaluated by the total antioxidant capacity (TRAP) in plasma at day 60 when compared to those at days 7 and 15 (Fig. 4D). Regarding of the assessment of oxidative stress markers of damage, fructose consumption increased systemic lipid peroxidation (TBARS) and protein oxidation (carbonyls) at the end of the protocol as compared to control normotensive and hypertensive animals (Fig. 4E,F).

## Discussion

The increasing prevalence of metabolic syndrome calls for research to design preventive or therapeutic strategies. We previously reported that increased sympathetic activity (15 days) preceded metabolic changes (60 days) in normotensive mice with fructose consumption^12^. More recent studies showed that changes in the baroreflex system preceded cardiovascular improvements (the arterial pressure reduction and bradycardia) observed in SHR rats after aerobic exercise training^16^. Given that recent studies show the potential of the nervous system to inhibit inflammation^17^ and oxidative stress, we hypothesize that alterations in the autonomic nervous system would precede metabolic and hemodynamic dysfunctions in genetically predisposed rats to hypertension with fructose overload.

Our findings showed that fructose overload associated with hereditary hypertension negatively affected the autonomic control of circulation, as demonstred by decreased baroreflex sensitivity since the beginning of protocol (7 days) in HF animals. The increase in inflamatory cytocines in the adipose tissse (TNF-α, IL-6 e IL-1β) and spleen (IL-1β) was detected at days 15 and 30 of protocol. We observed that this peak concurs with increased production of ROS (hydrogen peroxide-30 days), and decreased nitric oxide (NO) bioavailability (plasma nitrites) and SOD antioxidant enzyme (from day 15). Plasma triglyceride levels were increased at day 15, while plasma insulin levels were increased at day 30. At day 60, levels of antioxidant defense (SOD and TRAP) were decreased and correlated with the increased oxidative damage (lipid peroxidation and protein oxidation). Insulin resistance was also increased, together with a further increase in arterial pressure (at days 30 and 60) and reduced physical capacity (at day 60) in hypertensive animals with fructose overload.

Of note, 7 days of fructose overload are enough to induce autonomic dysfunction of baroreflex control of cardiovascular regulation in this experimental model of metabolic syndrome even without inducing any alteration in the metabolic profile (triglycerides, glucose, insulin and insulin resistance). This present study shows that hypertensive animals (with or without fructose) have a gradual increase in arterial pressure, along with reduced baroreflex sensitivity throughout the protocol. Both hypertensive experimental groups have lower baroreflex sensitivity for both bradycardic and tachycardic responses at days 30 and 60 as compared to that of the normotensive animals without fructose. However, fructose consumption in hypertensive rats enhanced the baroreflex impairment because hypertensive animals with fructose have a dysfunctional autonomic reflex of circulation at days 7 and 15 as compared to normotensive and hypertensive animals without fructose.

Previous studies showed decreased baroreflex sensitivity in hypertensive humans^18^, adult spontaneously hypertensive rats^16,19^ and normotensive adult rats with fructose overload^8^. However, our present study is the first report showing that fructose overload induced autonomic dysfunction from 7 days of treatment in spontaneously hypertensive rats. These results are important because the association of risk factors (hypertension plus fructose), mimics the metabolic syndrome, and seems to induce an early cardiovascular impairment. Since the baroreflex controls the cardiovascular reflex but also the sympathetic (inhibition) and parasympathetic (stimulation) activity^20^, the baroreflex dysfunction induced with fructose overload in hypertensive animals (37 days of life) in our study is associated with increased activity and/or decreased parasympathetic activity. Masson *et al*.^16^ reported increased central sympathetic activity in SHR rats (98 days old), while we previously reported increased sympathetic modulation in mice treated for 15 days with fructose (85 days of life)^12^. Furthermore, ovariectomized SHR rats with chronic consumption of fructose (135 days) had impaired heart rate and arterial pressure variability showing autonomic dysfunction^13,21^. However, these studies were performed in adult animals. By contrast, our study shows that adding a risk factor (fructose overload) in SHR rats induced autonomic dysfunction while growing, and earlier than in the SHR rats fed with standard diet.

Baroreflex dysfunction may be associated with vascular alterations and autonomic dysfunction. In this context, dysfunctions in vascular resistance have been previously reported in metabolic syndrome^22^ along with the individual pathologies associated with this syndrome^23,24^. In our present study, we showed that both hypertensive animals had decreased pressor responses to adrenergic stimulation induced by a dose of 2 µg/kg phenylephrine as compared to control normotensive animals.

We hypothesize that the different responses of normotensive Wistar and SHR rats to phenylphrine may be due to the expression of α1 adrenergic receptors in SHR rats^25^.

Our findings also show an exacerbated systemic depressor response to sodium nitroprusside at a 5 µg/kg dose during fructose consumption. This suggests that fructose consumption impaired the vascular relaxation, although statistically significant differences were only observed in normotensive animals without fructose. Simonet *et al*.^26^ reported that chronic treatment with L-NAME in SHR rats induced a vasodilation response to sodium nitroprusside exacerbated in the renal tissue. These results suggest that a more pronounced deficiency of NO bioavailability may lead to an exacerbated response to this drug. In this sense, our data show that fructose consumption decreased plasma nitrites in hypertensive animals as compared to those in control animals at days 15 and 30. These changes in vascular reactivity and in NO bioavailability may lead to exacerbated BRS impairment and to an additional increase in arterial pressure in the metabolic syndrome model used in this study. It should be noted that NO is a key mediator in endothelium-dependent vasodilation, having powerful anti-inflammatory and anti-thrombotic properties^27^.

Our results show that fructose overload increased TNF-α and IL-6 in the visceral white adipose tissue at days 15 and 30, and increased IL-1β at day 15. Fructose overload also increased in IL-1β in the spleen. Interestingly, all the inflammatory cytokines have similar levels in all groups at the end of protocol (60 days). Taken together, these findings suggest a marked transitory inflammatory signaling at days 15 and 30. We analyzed the inflammatory factors in the visceral white adipose tissue and the spleen, the two key tissues that release cytokines into systemic circulation. Recent studies demonstrated that the afferent vagal nerve attenuates systemic inflammation by inhibiting the production of inflammatory cytokines in the spleen^11,17^. Our findings suggest that the baroreflex sensitivity impairment (strongly related to sympathovagal cardiovascular dysfunction) observed early in fructose-treated hypertensive animals (7 days of protocol) may increase the production of inflammatory mediators (at days 15 and 30) in the adipose tissue and the spleen.

Studies assessing the chronic effects of a fructose-rich diet in adult animals reported increased TNF-α, IL-1β and IL-6 in adult male and female normo and hypertensive rats^13,28,29^. Of note, increase in TNF-α levels was associated to insulin resitance^30^. In this study fructose overload in hypertensive rats, despite unchanged expressively body weight, increased plasma triglycerides and insulin, and reduced sensitivity to insulin, along with increased number of inflammatory mediators in the adipose tissue and spleen. These changes in our experimental model reflect the key features of metabolic syndrome^3,9,15^.

In previous studies inflammation increases oxidative stress, which, in turn, can cause cell dysfunctions with hypertension, and contribute to disease progression^31^. Fructose consumption consistently presented the greatest systemic concentration of hydrogen peroxide (at day 30 and 60), decreased SOD activity (at days 15, 30 and 60) and TRAP (at days 7 and 15), increased lipid peroxidation and protein oxidation (at day 60). Previous studies suggest that systemic and tissue unbalanced redox status appeared early in this experimental model and may play a role on cardiometabolic disorders^32–35^. Our results concur with previous studies reporting that a 21 day fructose rich diet increased tryglicerides, plasma insulin, and oxidative stress without changing glucose levels^28,36^.

We recently reported that fructose overload (for 19 weeks) induced cardiovascular autonomic dysfunction, inflammation (increased TNFα and decreased IL-10), increased lipid peroxidation and unbalanced glutathione redox balance in cardiac tissues in ovariectomized female SHR rats. We also reported increased triglycerides and insulin resistance and additional increase in AP^13^. In this present study, the SHR rats have increased diastolic, systolic and mean arterial pressure throughout the protocol (60 vs. 7, 15, 30 days). Hypertension in SHR rats starts at 35 days old, but environmental factors may affect disease progression. Accordingly, in this study SHR rats started chronic fructose consumption at 30 days old. Fructose consumption induced an additional increase in arterial pressure as compared to control hypertensive animals, but only after 30 and 60 days of the fructose overload. Moreover, both normo and hypertensive control animals have decreased HR at days 15, 30 and 60 as compared to those of day 7 (37 days old). In previous studies, the increased HR in the first days after birth is probably associated to the positive chronotropic effect of the sympathetic nervous system on the HR of rats^37^. In this sense, fructose consumption seems to maintain the chronotropic effect of the sympathetic nervous system, since reduced HR was not observed at any point of the protocol, unlike the HR changes of normo and hypertensive control animals. The baroreflex dysfunction observed from day 7 suggests a greater role for sympathetic participation in cardiovascular regulation of fructose-overloaded SHR rats.

In summary, fructose consumption impairs baroreflex sensitivity may inducing inflammatory cytokines in the spleen and the adipose tissue. This would lead to an unbalanced systemic redox state producing insulin resistance and increasing arterial pressure, together with reduced functional capacity, well-known features of the metabolic syndrome.

## Future Perspectives

Baroreflex impairment, which reflects primarily a disarrangement in the vagal efferent component of this reflex, is strongly associated with sympathetic overactivity, activation of renin-angiotensin system, inflammation, oxidative stress, increased arterial pressure variability and end-organ damage^8,9,13,16,17,38^. Additionally, this condition is important and common characteristic of hypertension and is related to increased mortality risk in hypertensive subjects, renal failure and post infarction patients^18,39,40^. Our data indicated that the baroreflex is the first mechanism affected by the association of hypertension and fructose overload and precedes the increase in inflammation and oxidative stress in target tissues, later on inducing arterial pressure increase and insulin resistance. This time course mechanisms offers the possibility to understand the integrative cardiometabolic dysfunctions in several diseases and to develop new and appropriate therapeutic targets, mainly focusing on the improvement of cardiovascular autonomic control of circulation.

## Methods

30 days old male SHR and Wistar rats were obtained from the Animal Facility of the Universidade Nove de Julho. The rats were randomly divided into 3 groups: control normotensive (C, n = 24), hypertensive (H, n = 24) and hypertensive undergoing fructose overload (HF, n = 24). Fructose overload in drinking water (D-fructose, 100 g/L) was initiated at 30 days of life in Fructose consumption. Animals from C and H groups received standard laboratory chow and water ad libitum. The evaluations were performed in 6 rats for each group after 7, 15, 30 and 60 days of fructose or water consumption. The Ethics Committee of Sao Paulo University approved all surgical procedures and protocols (Protocol 035/12) and they were performed in accordance with the National Institutes of Health -Guide for the Care and Use of Laboratory Animals.

### Functional Capacity

All animals were adapted to a motor treadmill (Imbramed TK-01, Brazil) (0.3 km/h; 10 min/day) for 3 days previous to the exercise test. To perform functional capacity evaluation one day before the last day of protocol, the rats underwent a maximum running test with increasing velocity (0.3 Km/h) every 3 minutes until fatigue, as described in detail in a previous study^41^.

### Blood triglyceride and glucose

In the last day of protocol, before catheterization, triglyceride and blood glucose concentrations were measured (Accucheck and Accutrend, Roche) after 4-hour fasting.

### Insulin tolerance test

Following hemodynamic evaluation, insulin tolerance test was performed to obtained the constant rate for blood glucose disappearance (KITT) as previous described^7,13^.

### Cardiovascular and autonomic assessments

In the last day of protocol, catheters were implanted for drugs administration and direct measurements of AP (20 min) in conscious animals as previous described^13^.

Phenylephrine and sodium nitroprusside were injected sequentially to obtain AP responses ranging from 5 to 40 mm Hg. Baroreflex sensitivity was assessed by a mean index relating changes in HR to changes in MAP, allowing a separate analysis of gain for reflex bradycardia and reflex tachycardia as described elsewhere^13,19,21^.

Vascular reactivity was determined by the AP changes following phenylephrine (2 μg/kg) and sodium nitroprusside (5 μg/kg) injections. Baseline values and peak changes of MAP for each given dose were considered in data analysis.

### Plasma nitrites

The animals were killed one day after hemodynamic evaluations, and the white adipose tissue, spleen and plasma were immediately collected for analysis. In order to determine the plasma nitrites (NO^−^_2_) we used the Griess reagent^13^.

### Insulin determination

Insulin concentration was measured in plasma of fasting animals (4 hours) by immunoenzymatic test (ELISA) using a commercial kit (EZRMI-13K/Rat/Mouse insulin, Merck Millipore, USA). Test sensitivity was 0.2 ng/ml. Using a microplate reader, absorbance was measured at 450 nm.

### Inflammatory mediators in spleen and in visceral white adipose tissue

IL-6, IL-1β and TNF-α levels were determined in the visceral white adipose tissue and spleen using a commercially available ELISA kit (R&D Systems Inc.) as previous described^13^.

### Oxidative stress profile in plasma

The techniques for determination of oxidative stress profile were previously described in details^13^. Hydrogen peroxide concentration was assessed based on the horseradish peroxidase- (HRPO) mediated oxidation of phenol red by H_2_O_2_^42^.

The quantification of SOD activity was based on the inhibition of the reaction between O2˙^−^ and pyrogallol^43^. CAT activity was determined by measuring the decrease in H_2_O_2_ absorbance at 240 nm^44^. The TRAP was measured using 2,2′-azo-bis (2-amidinopropane) (ABAP) and luminol in a liquid scintillation counter^45^.

The lipid peroxidation was evaluated by thiobarbituric reactive substances (TBARS)^46^. The determination of protein oxidation was determined by carbonyls technique^47^.

### Statistical analysis

Data are expressed as mean ± SEM. In order to evaluate data homogeneity, the Levene test was used and to compare groups, we performed a one-way analysis of variance followed by the Student-Newman-Keuls test. Significance level was defined at p ≤ 0.05.
